# Low-level viraemia: An emerging concern among people living with HIV in Uganda and across sub-Saharan Africa

**DOI:** 10.4102/ajlm.v11i1.1899

**Published:** 2022-10-20

**Authors:** Nicholus Nanyeenya, Noah Kiwanuka, Damalie Nakanjako, Gertrude Nakigozi, Simon P.S. Kibira, Susan Nabadda, Charles Kiyaga, Isaac Sewanyana, Esther Nasuuna, Fredrick Makumbi

**Affiliations:** 1 Department of Epidemiology and Biostatistics, School of Public Health, Makerere University College of Health Sciences, Kampala, Uganda; 2 Ministry of Health Central Public Health Laboratories, Kampala, Uganda; 3 Department of Medicine, School of Medicine, Makerere University College of Health Sciences, Kampala, Uganda; 4 Rakai Health Sciences Project, Masaka, Uganda; 5 Department of Community Health and Behavioral Sciences, School of Public Health, Makerere University College of Health Sciences, Kampala, Uganda; 6 Infectious Diseases Institute, Makerere University College of Health Sciences, Kampala, Uganda

**Keywords:** HIV/AIDS, low-level viraemia, viral load testing, non-suppression, virologic failure

## Abstract

Attaining viral load (VL) suppression for over 95% of the people living with HIV on antiretroviral therapy is a fundamental step in enabling Uganda and other sub-Saharan African countries to achieve global Sustainable Development Goal targets to end the HIV/AIDS epidemic by 2030. In line with the 2013 World Health Organization recommendations, several sub-Saharan African countries, including Uganda, use a threshold of 1000 HIV viral RNA copies/mL to determine HIV viral non-suppression. The United States Centers for Disease Control and Prevention and the International Association of Providers of AIDS Care deem this threshold very high, and hence recommend using 200 copies/mL to determine viral non-suppression. Using 1000 copies/mL as a threshold ignores people living with HIV who have low-level viraemia (LLV; HIV VL of at least 50 copies/mL but less than 1000 copies/mL). Despite the 2021 World Health Organization recommendations of using intensive adherence counselling for people living with HIV with LLV, several sub-Saharan African countries have no interventions to address LLV. However, recent studies have associated LLV with increased risks of HIV drug resistance, virologic failure and transmission. The purpose of this narrative review is to provide insights on the emerging concern of LLV among people living with HIV receiving antiretroviral therapy in sub-Saharan Africa. The review also provides guidance for Uganda and other sub-Saharan African countries to implement immediate appropriate interventions like intensive adherence counselling, reducing VL thresholds for non-suppression and conducting more research to manage LLV which threatens progress towards ending HIV by 2030.

## Introduction

By the end of 2020, an estimated 37.7 million people were living with HIV/AIDS; two-thirds (67%) were living in sub-Saharan Africa, and 27.5 million were accessing antiretroviral therapy (ART).^1^ The increased access to ART has necessited the monitoring of its efficacy among people living with HIV, hence the increased scale-up of HIV viral load (VL) monitoring.

As the world strives to control the HIV epidemic, attaining VL suppression for over 95% of people living with HIV on ART is a fundamental step in enabling Uganda, and other sub-Saharan African countries, to achieve the global targets of ending the HIV/AIDS epidemic by 2030, as stipulated in target 3.3 of the Sustainable Development Goal 3.^1,2,3^ HIV viral non-suppression is defined as HIV RNA viral copies equal to or greater than 1000 copies/mL as recommended by the World Health Organization (WHO).^4^ This is also an indication of HIV virologic treatment failure in Uganda, and several other sub-Saharan African countries like Kenya, Zambia and Sierra Leone, among others.^5^ Low-level viraemia (LLV) defined as having an HIV VL of at least 50 copies/mL but less than 1000 copies/mL (≥ 50 copies/mL to < 1000 copies/mL) is considered as being virally suppressed. Despite the recent 2021 WHO recommendation to offer intensive adherence counselling (IAC) to people living with HIV on ART with LLV,^6^ no special intervention is given to people living with HIV with LLV in Uganda and several other sub-Saharan African countries; yet, LLV has been associated with various clinically poor outcomes like HIV drug resistance and virologic failure.^7,8^ This narrative review was completed in January 2022, and the reviewed articles were published from March 2000 to June 2021. Several databases including Google Scholar, PubMed, and Science Direct were searched using search terms like ‘low-level viraemia’, ‘viral load testing’, ‘non-suppression threshold’ and ‘virologic failure’. This review aims at guiding Uganda and other sub-Saharan African countries to take immediate appropriate interventions to manage LLV.

## Antiretroviral therapy monitoring

Highly active ART has been the springboard in the management and control of HIV/AIDS over the years; the goal of highly active ART is to lead to HIV viral suppression with an increase in the body immunity function.^4^ Globally, access to ART has increased, thereby improving the HIV treatment outcomes,^1^ and this has been a great milestone in achieving epidemic control. In Uganda, an estimated 1.2 million (out of 1.4 million) people living with HIV were on ART by 2019.^9^ As the efforts to scale-up people living with HIV on ART continue, there is an inevitable need to monitor the efficacy of ART among people living with HIV^10^ and several methods of ART monitoring are recommended by WHO. These include patient monitoring involving clinical events and adherence monitoring, immunological monitoring involving CD4 cell count, and virologic monitoring comprising HIV VL testing.^11, 12^

### Virologic monitoring

Virologic monitoring which comprises HIV VL testing is the preferred way of monitoring treatment outcomes in people living with HIV on ART, and WHO recommends use of a threshold of 1000 copies/mL to determine VL non-suppression, indicative of either poor drug adherence or virologic failure.^6^ However, the United States Centers for Disease Control and Prevention and International Association of Providers of AIDS Care recommend a threshold of 200 copies/mL for VL non-suppression.^13, 14^

A number of sub-Saharan African countries initiated the scale-up of VL testing from 2014 to 2015, following the WHO 2013 recommendation.^15, 16^ Uganda began scaling up HIV VL testing for all eligible people living with HIV on ART in 2014.^16^ Upon initiation of ART, the first VL test is done for people living with HIV at six months, and then at 12 months, after which it is repeated annually for suppressed adults, and every six months for suppressed children and adolescents. People living with HIV with a VL result below 1000 copies/mL have a suppressed VL and are routinely given adherence counselling in which they are encouraged to continue with ART, and no other intervention is given.^5^ A decreased VL is associated with better clinical outcomes and slowed disease progression,^17^ and is also associated with reduced HIV incidence at community level.^17, 18^ However, there is limited data about the cut-off of viral copies at which the slowed disease progression and reduced HIV incidence at community level happens.

People living with HIV/AIDS who have been on ART for at least six months with a VL of 1000 copies/mL or more are non-suppressed. These people are offered monthly IAC sessions for three months. After the IAC sessions, a VL test is repeated in the fourth month to determine whether they have achieved VL suppression.^5, 6, 12^ If the repeat VL result after IAC is still non-suppressed, this is considered as virologic failure provided non-adherence to ART is ruled out. A switch committee is then convened to discuss switching the patient to another ART line.^5^ Several predisposing factors like poor ART adherence, unawareness about the ART benefits and other existing chronic illness, among others, have been associated with VL non-suppression.^19, 20, 21^ People living with HIV on ART with non-suppressed VL are at increased risk of faster progression to AIDS, which is also associated with poor clinical outcomes.^21, 22^

Tremendous progress has been achieved by several sub-Saharan African countries in establishing comprehensive VL testing programmes^23^; for instance, in Uganda, the number of annual VL tests has steadily increased annually from 16 411 VL tests (2% of people living with HIV on ART) in 2014 to 1 332 335 VL tests (95% of people living with HIV on ART) in 2020.^24^ Challenges affecting the scale-up of VL testing, like suboptimal sample transportation and results delivery, limited human resource in health facilities, unawareness about VL testing among HIV healthcare providers and people living with HIV, protracted procurement processes, and poor adherence to national and WHO VL testing guidelines, have limited full coverage of VL testing in the country.^23, 25^ The other key important issue to note with the introduction of VL testing is LLV.

## Low-level viraemia

### Definition and risk factors

The WHO defines LLV as a low but detectable VL that is, viraemia (≥ 50 copies/mL to < 1000 copies/mL).^26^ People living with HIV/AIDS on ART with viraemia ≥ 50 copies/mL to < 1000 copies/mL are virally suppressed, but have LLV.^5, 27^ Previous studies have associated LLV with baseline CD4 + T cell counts below 200 cells/mm^3^, ART duration longer than 60 months, ART regimens comprising of zidovudine, lamivudine and nevirapine (aOR: 2.26, *p* < 0.001) or didanosine-based regimen, and existing subtype B′ infection.^27, 28^

### Justification for using a threshold of 1000 copies/mL for viral load non-suppression

The use of 1000 copies/mL as a threshold for determining VL non-suppression in sub-Saharan African countries including Uganda, has generated enormous debate over the years, since it can lead to the accumulation of people living with HIV on ART categorised as suppressed VL, but having LLV.^29^ The recommendation by WHO to use this threshold of 1000 copies/mL^30^ was based on a public health perspective where dried blood spot samples were used instead of plasma as a specimen type for HIV-1 VL testing, facilitate the decentralisation of specimen collection and can increase access to VL testing in resource-limited settings like Uganda due to their cost-effectiveness, as compared to plasma.^31^ Unfortunately, the performance of dried blood spot samples for VL testing is lower, when compared to the gold standard sample type plasma, with a low sensitivity and specificity to detect treatment failure, and favours using a threshold of 1000 copies/mL.^29, 32^

Slowed disease progression has been demonstrated in people living with HIV on ART with a VL of less than 1000 copies/mL,^17, 33^ though the exact threshold at which LLV predicts disease progression varies, and remains debatable.^34^ Furthermore, a reduced risk of HIV transmission for people living with HIV on ART with VL results less than; 1000 copies/mL,^35^ 1500 copies/mL,^36^ and less than 1700 copies/mL,^37^ has been shown. No HIV transmission risks have been demonstrated below 400 copies/mL,^38^ but the range of viraemia between 400 copies/mL and 1000 copies/mL at which HIV transmission occurs, still remains unclear.^39^

### Prevalence and effects

Sustained VL suppression has been shown to reduce the occurrence of HIV drug resistance among people living with HIV on ART and also leads to improved treatment outcomes.^40, 41, 42^ However new emerging resistance mutations have been demonstrated in people living with HIV on ART with LLV,^7,8^ where persistent LLV has been associated with increased risk of virologic failure^43^; which is also associated with increased risk of adverse treatment outcomes.^44^

For the case of the sub-Sahara African region, 19.3% of the participants had LLV while 7.8% of the participants had persistent LLV in a study, which aimed to evaluate virologic failure and its predictors in four African countries including Uganda, Kenya, Tanzania and Nigeria.^45^ Furthermore, 57.5% of participants with persistent LLV (plasma HIV RNA > 50 copies/mL at two consecutive visits) in this study would later have confirmed virologic failure. However this study did not examine HIV drug resistance, which could be a key driver of virologic failure (failure of people living with HIV with a non-suppressed VL to suppress after three sessions of IAC, and poor drug adherence has been ruled out), and also assessment of drug adherence was mainly through self reports which could have been affected by social desirability and recall bias.^45^

Furthermore, in South Africa, LLV occurred in 23% of people living with HIV on ART with an incidence of 11.5 per 100 person-years of follow-up (95% confidence interval: 11.4–11.7), during first-line ART. In this study, LLV was associated with increased hazards ratios of virological failure and the subsequent switch to second-line ART, as compared with a non-dectectable VL of less than 50 copies/mL; and the risk of virological failure was increased more with increased ranges of LLV.^7^ This study did not look at HIV drug resistance testing results in LLV, because they were not available.

In another South African study looking at HIV viraemia and mother-to-child transmission risk after ART initiation in pregnancy in Cape Town, the risk of early HIV vertical transmission was greatly associated with VL at delivery, with noted risks of 0.25%, 2.0% and 8.5% among women with VL < 50 copies/mL, 50 copies/mL – 1000 copies/mL and > 1000 copies/mL at delivery, respectively (*p* < 0.001). This implies that women with LLV at delivery had eight times the risk of HIV vertical transmission, as compared to women with a non-detectable VL < 50.^46^

In Malawi, a study was conducted that comprised 1274 mothers and described VL suppression among HIV-positive mothers at 4–26 weeks postpartum, the factors associated with VL suppression, and the impact of VL suppression levels on mother-to-child transmission. This study indicated that 8.7% of these HIV-positive mothers had LLV and were more likely to be adolescents, who had been on ART for less than six months, with suboptimal adherence. LLV was associated with 7.0% risk of mother-to-child transmission, as compared to 0.9% risk for mothers with a non-detectable VL.^47^

Elsewhere, HIV drug resistance was detected in about 30.0% of people living with HIV with a VL test having the first episode of LLV in British Columbia; and these patients had an increased risk of developing virologic failure, compared to those without HIV drug resistance.^48^ Another study indicated that there were 44.0% accumulated resistance mutations in 47 people living with HIV on ART with two or more episodes of LLV, and surprisingly the median viraemia was 267 copies/mL; and virologic failure followed in 16.0% of these people living with HIV.^49^ In the United Kingdom, 30.0% of people living with HIV with LLV acquired at least one drug resistant mutation^50^; and major resistant mutations were detected in 12.7% of people living with HIV with LLV.^51^

### Proposed interventions

Reduced drug adherence has been associated with residual LLV,^52^ and this implies that interventions to enhance treatment adherence could be offered to people living with HIV on ART with LLV to achieve a non-detectable VL, which is the recommended target of ART in various international guidelines.^53, 54^ In the recent 2021 WHO consolidated guidelines, IAC has been recommended to be offered to people living with HIV with LLV,^6^ though most sub-Sahara African countries have not yet started offering this intervention. Furthermore, there is no data in Uganda and other sub-Sahara African countries to determine whether IAC can be effective in achieving a non-detectable VL among people living with HIV on ART with LLV. Few sub-Sahara African countries like South Africa^7, 46^ and Malawi^47^ have undertaken research to understand LLV and its implications, which has created a knowledge gap about the subject, thereby affecting key policy decisions in the region.

## Conclusion

There is an increasing frequency of LLV among people living with HIV on ART in Uganda and across other sub-Sahara African countries. This LLV is associated with increased risks of HIV drug resistance and virologic failure which can affect the efficacy of ART, and lead to accelerated HIV disease progression. With the advancing global targets to end the HIV epidemic, reduce transmission rates, and improve the clinical outcomes of the people living with HIV on ART, there is an inevitable need to re-assess the use of the threshold of 1000 copies/mL to determine VL non-suppression, and to establish strategies to address the rising proportions of people living with HIV on ART with unmanaged LLV in the region. More research about the association between LLV and drug resistance and virologic failure in sub-Sahara Africa also needs to be done.
